# Sensitivity of Rabbit Ventricular Action Potential and Ca^**2+**^ Dynamics to Small Variations in Membrane Currents and Ion Diffusion Coefficients

**DOI:** 10.1155/2013/565431

**Published:** 2013-10-07

**Authors:** Yuan Hung Lo, Tom Peachey, David Abramson, Andrew McCulloch, Anushka Michailova

**Affiliations:** ^1^Department of Bioengineering, PFBH 241, University of California San Diego, 9500 Gilman Drive, La Jolla, CA 92093-0412, USA; ^2^Faculty of Information Technology, Monash University, Clayton, VIC 3800, Australia

## Abstract

Little is known about how small variations in ionic currents and Ca^2+^ and Na^+^ diffusion coefficients impact action potential and Ca^2+^ dynamics in rabbit ventricular myocytes. We applied sensitivity analysis to quantify the sensitivity of Shannon et al. model (Biophys. J., 2004) to 5%–10% changes in currents conductance, channels distribution, and ion diffusion in rabbit ventricular cells. We found that action potential duration and Ca^2+^ peaks are *highly sensitive* to 10% increase in L-type Ca^2+^ current; *moderately influenced* by 10% increase in Na^+^-Ca^2+^ exchanger, Na^+^-K^+^ pump, rapid delayed and slow transient outward K^+^ currents, and Cl^−^ background current; *insensitive* to 10% increases in all other ionic currents and sarcoplasmic reticulum Ca^2+^ fluxes. Cell electrical activity is strongly affected by 5% shift of L-type Ca^2+^ channels and Na^+^-Ca^2+^ exchanger in between junctional and submembrane spaces while Ca^2+^-activated Cl^−^-channel redistribution has the modest effect. Small changes in submembrane and cytosolic diffusion coefficients for Ca^2+^, but not in Na^+^ transfer, may alter notably myocyte contraction. Our studies highlight the need for more precise measurements and further extending and testing of the Shannon et al. model. Our results demonstrate usefulness of sensitivity analysis to identify specific knowledge gaps and controversies related to ventricular cell electrophysiology and Ca^2+^ signaling.

## 1. Introduction

Several ionic models have been developed to investigate the subcellular mechanisms regulating excitation-contraction coupling (ECC) in rabbit ventricular cardiomyocytes [1–7]. In 2004, Shannon and colleagues published a detailed model for Ca^2+^ handling and ionic current that accurately represents sarcoplasmic reticulum (SR) Ca^2+^-dependent release and simulates basic ECC phenomena. This model was the first to include (1) the subsarcolemmal Ca^2+^ compartment to the other two commonly formulated cytosolic Ca^2+^ compartments (junctional and bulk, [8]), (2) the variations in the locations of ion transporters throughout the cell surface membrane, (3) Ca^2+^ and Na^+^ transport between the subcellular compartments, and (4) Na^+^ buffering inside. Latest studies extended further the Shannon et al. ionic model in rabbit ventricular cells. Mahajan and colleagues modified L-type Ca^2+^ current and Ca^2+^ cycling formulations, based on new experimental patch-clamp data, and used the updated model to investigate the mechanisms regulating ventricular tachycardia and fibrillation [4, 5]. Morotti et al. improved Mahajan's et al. model of rabbit ventricular *I*_Ca_ and examined the relative contributions of voltage- and Ca^2+^-dependent inactivation to total current inactivation [7]. Aslanidi et al. determined the functional role of each ionic channel current in modulating the action potential (AP) shape from different locations in rabbit ventricular wall [6]. Recently, using parameter sensitive analysis, Romero et al. [9] identified also key subcellular factors involved in the electrical signal generation and Ca^2+^ signal regulation in two rabbit cell models [1, 4]. Systematically characterizing AP properties, Ca^2+^ and Na^+^ dynamics, and their rate dependence on ±15%, ±30, and ±45% variations in the main transmembrane currents' conductance they found that APD is significantly modified by most repolarization currents and that steady-state Ca^2+^ levels are strongly dependent on *I*_Ca_, *I*_NCX_, and *I*_NaK_ currents. Important limitation of these “common pool models”, however, is that the subcellular compartment geometries (junctional cleft, submembrane space, cytosol, and SR) were simplified to allow prediction of total Ca^2+^ concentration ([Ca](t)) in the compartment of interest only.

A few reaction-diffusion models that seek to incorporate the effects of idealistic or more realistic subcellular and whole-cell geometries and to compute the spatial distributions of [Ca] in cardiac, skeletal or smooth muscle cells have been published also [10–19]. Recently, the contributions of structural heterogeneities to local and global Ca^2+^ signals in rabbit ventricular myocytes with realistic *t*-tubule microanatomy have been investigated [20]. Limitations of these spatial models, due to complexities, are that the cell electrophysiology was simplified, the AP dynamics was not reproduced, and the effects of intracellular Na^2+^ diffusion and buffering on ECC were not investigated.

Taken together the published computational models have provided important insights into the underlying mechanisms regulating intracellular Ca^2+^ dynamics and electrophysiological behavior in rabbit ventricular cells under control and certain pathological conditions. It remains, however, poorly understood how small variations in ion currents conductance and distribution and small changes in Ca^2+^ and Na^+^ transport between cellular subdomains affect important cellular biomarkers (i.e., AP shape, [Ca]_*i*_, [Ca]_SL_, and [Ca]_jct_). In this study, we applied sensitivity analysis and used parameter estimation tools to investigate these questions. The sensitivity of each biomarker to 5%–10% changes in ionic currents properties and Ca^2+^ and Na^+^ diffusion coefficients were quantified using the Shannon et al. model. Validation of simulation results was performed by a comparison to experimental data in rabbit ventricular myocytes. This study identifies new experimental data required for better understanding of rabbit's electrophysiology and demonstrates the importance of sensitivity analysis as a powerful method for systematic and in-depth validation of AP models.

## 2. Methods

### 2.1. Electrophysiological Model in Rabbit Ventricular Myocytes

**Figure 1:**
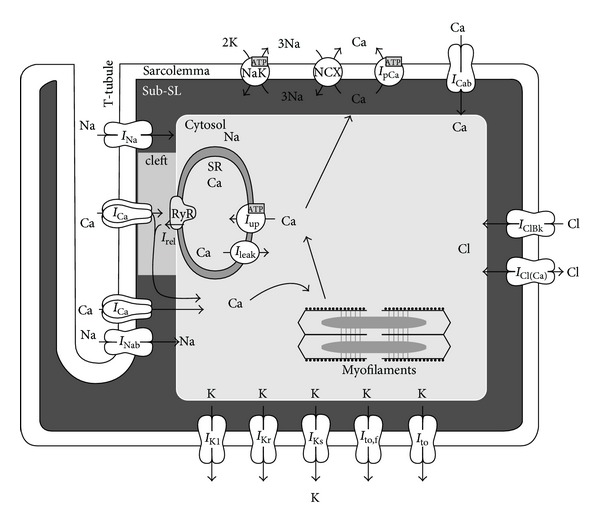
Schematic diagram illustrating the four subcellular compartments, electrophysiology, and Ca^2+^ dynamics in the Shannon et al. model in rabbit ventricular myocytes [1]. See Glossary and Tables 1–3 for the notations of the parameters used throughout the study.

A diagram describing the arrangement of subcellular compartments, ionic currents and pumps, and intracellular Ca^2+^ fluxes included in the Shannon et al. electrophysiological model is shown in Figure 1.

 The Shannon et al. model was adopted without changes and recent MatLab version of the code has been uploaded (http://somapp.ucdavis.edu/Pharmacology/bers/). The model behavior at 1 Hz pacing frequency is consistent with experimental measurements for AP-shape and global cytosolic Ca^2+^ concentrations ([Ca]_*i*_) changes under control conditions [1].

### 2.2. Sensitivity Analysis Tools

Recently, several sensitivity analysis and parameter estimation tools and methodologies have been developed with a specific aim to assess the robustness of the cardiac cell models' parameters, including (1) the parameter variability analysis which examine the effects of varying one parameter in time [21], (2) techniques which assess the significance of parameters using multivariable regression over randomized set of input parameters values [22], and (3) global sensitivity analysis tool which implements the Morris screening algorithm [23] within the Nimrod platform [24]. In this study, we explored two methodologies [22, 23] and compared each method potential in evaluating parameter-output correlations in the Shannon et al. electrophysiological model.

#### 2.2.1. Partial Least Squares (PLS) Regression

PLS is a statistical method that creates a linear regression model to predict a set of dependent variables (i.e., model outputs) from a set of independent variables (i.e., model inputs) [22, 25]. PLS is particularly useful when inputs are highly correlated (i.e., multicollinear) and when there are more inputs than outputs. In regression analysis, if two highly correlated effects are measured, the more dominant effect will falsely enhance the weaker effect, leading to inaccurate sensitivity information, and misinform truly significant parameter effects [25]. Since PLS retains good predictive power when working with multicollinear data, myocyte models containing multiple correlated variables can be analyzed using PLS.

#### 2.2.2. Nimrod Tool Family 

*Nimrod* uses a simple declarative modeling language to define parametric experiments, and it automates the tasks of formulating, running, monitoring, and collecting results from multiple individual experiments. Nimrod is not a single tool. It incorporates Nimrod/G, component that distributes computations to the recourses [24, 26]; Nimrod/O, a component that searches for “good” solutions using nonlinear optimization algorithms [27]; Nimrod/E, component that helps evaluating which parameter settings are important using the experimental design [28]. *Nimrod/G* is designed to assist scientists in performing studies, using concurrent execution on a cluster of processors or the resources of a computational grid. The user prepares a “*plan file*” which specifies the experiment [24]. A plan file is expanded into a “*run file*” which lists the appropriate parameter combinations required for execution. The Nimrod/G core runs on a processor called the “*root node*” whereas individual jobs are executed on “*remote nodes.*” Nimrod/G handles the file transfers required for the remote nodes, execution of computational tasks, and transfer of results back to the root node. The number of concurrent jobs is limited only by the number of processors available. Thus the user may achieve high concurrency without modifying the executables and without concern for grid specific details. *Nimrod/O* optimizes the numerical outputs of computational models. It provides a range of optimization algorithms and leverages Nimrod/G to perform batches of concurrent evaluations. The user prepares a “*schedule file*,” which, like the Nimrod/G plan file, specifies the parameters, their ranges, and the tasks required for execution of the model. But it also specifies the optimization algorithms to be used and the settings for the algorithms. Nimrod/O performs multiple searches for an algorithm and multiple algorithms, all in parallel. 

#### 2.2.3. Nimrod/E and Fractional-Factorial Design (FFD)

It is often of interest for researchers to identify variable interactions because they may provide physiological insights into cell dynamics. While colinearity in multiple linear regressions is problematic and can lead to inaccurate predictions, Nimrod/E is able to circumvent this problem and successfully identify both main effects and multifactor interactions [28]. Similar to PLSR, FFD attempts to create a linear model to explain the data generated from the model; it measures and ranks main effects and two-level interactions of input variables with outputs via experimental design. However, the method chooses only those jobs that generate the most significant results by ignoring high-level interactions with little influence, thereby saving a lot of computation time. From the resulting linear model effect coefficients can also be extracted that tells the correlation between parameters and model outputs. For more complete view of successes and failures in modeling pursuits of Nimrod toolsets, refer to Abramson and colleagues papers [24, 26–28].

### 2.3. Parameter Sensitivity Analysis

**Figure 2:**
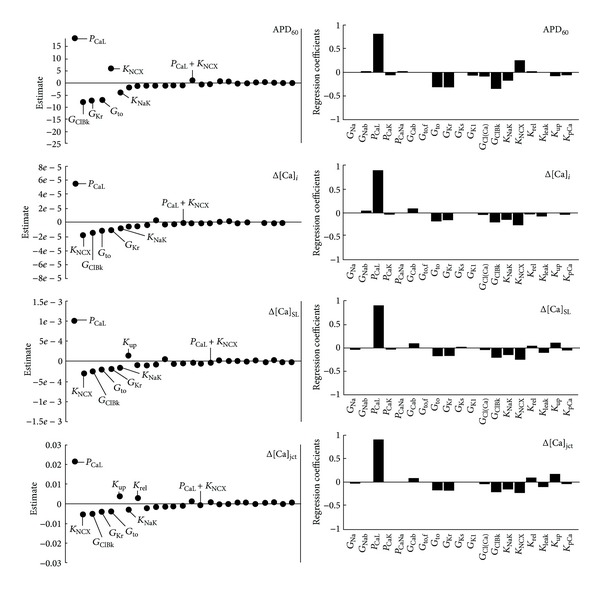
Parameter sensitivity analysis of APD_60_, Δ[Ca]_*i*_, Δ[Ca]_SL_, and Δ[Ca]_jct_ sensitivities toward 10% increases in maximal transporters' conductance and permeability. Positive value indicates direct relationship between parameter and output (e.g., enhanced L-type Ca^2+^ permeability prolongs APD_60_), and negative value indicates indirect relationship between parameter and output. Nimrod/E-Lenth plots (left column), PLS-bar plots (right column). Nimrod/E analysis produced similar qualitative results as PLS analysis.

To perform sensitivity analysis, the Shannon et al. MatLab code was modified to extract the desired model outputs. Under basal conditions or by varying one parameter at a time AP and Ca^2+^ transients appeared stable (after 6–8 s until 8 min) at stimulus frequency of 1 Hz. For this reason we recorded and analyzed AP and Ca^2+^ signals ([Ca]_*i*_, [Ca]_SL_, [Ca]_jct_) at 9-10 s of every simulation. In all numerical experiments (0 ≤ *t* ≤ 8 min) [K]_*i*_ remained unchanged while the variations in [Na]_*i*_ were small (ranging from 20 *μ*M to 100 *μ*M) with insignificant effects on calculated AP and Ca^2+^ traces at 6–8 s. The APD_60_ and delta [Ca]_*j*_ (Δ[Ca]_*j*_, *j* = *i*, SL,  jct) quantities were used to analyze the model sensitivity. Δ[Ca]_*j*_ is defined as the difference between peak Ca^2+^ concentration in the particular compartment and the diastolic Ca^2+^ concentration of 0.1 *μ*M. The modified code was uploaded onto Nimrod/E toolset and was executed repeatedly using permutations of parameter sets that were ±10% or ±5% of default values (see the appendix, Tables 1–3). Then Nimrod/E fractional-factorial analysis and PLS were used to analyze the effects of single parameter changes. In addition, Nimrod/E allowed examining the two-level parameter interactions on model outputs. In the Results section the sensitivities of selected biomarkers to variations of parameters are displayed graphically as either “*Lenth plots*” in Nimrod/E, or as “*bar plots*” in PLS. Before performing PLS regression analysis to obtain sensitivity values, *z*-scores of input and output matrices were calculated using MatLab's *z*-score function; that is, *z* = (*x* − mean(*x*))/std(*x*). Each *z*-score value was computed using the mean and standard deviations along each column of the matrices. The columns of matrices have mean zero and standard deviation one. The PLS regression coefficients, or sensitivity values (see Figures 2, 4, and 6), indicate how changes in input parameters lead to changes in outputs. Examining these numbers allows for an assessment of the relative contributions of the various parameters. The sensitivity values in the “*bar plots*” could be interpreted quantitatively as follows. Because input and output matrices are mean-centered and normalized to standard deviations computed column by column, each sensitivity value is defined relatively to the relevant deviations. For instance, if the regression coefficient for the input *P*_CaL_ and the output APD_60_ is 0.5, then when *P*_CaL_ is one standard deviation greater than the mean, APD_60_ will increase by half a standard deviation. Conversely, if the value is −0.5, then when *P*_CaL_ is one standard deviation greater than the mean, APD_60_ will decrease by half a standard deviation, and vice versa. The *estimate* values in the “*Lenth plots*” (*y*-axis) provide a qualitative overview of the inputs' relative effects on outputs and offer a comparison to the PLS-bar graphs. Changing the number of parameters studied slightly varied the magnitude of “*regression coefficients*” of bar plot and “*estimate*” of Lenth plot, but the relative effects and overall parameter-to-output relationships remained constant. The effects of sample sizes on model predictive efficacy by randomly picking subsets from sample pool, ranging from 15, 20, 50, 100, 200, and 500 to 1000 trials was examined. The adjusted *R* ^2^ values (*coefficient of determination*, *quantifying the explanatory capacity of the regression analysis*) were calculated and averaged. At the low end of 15 samples, the regression model explained 90.8 ± 2.4% of the variance. At the high end of 1000 samples, the model explained 96.4 ± 0.3% of the variance. This relatively low decrease in prediction efficacy suggests that sample sizes do not significantly change the qualitative information obtained from the experiments. Considering this statistical analysis, the results were not included.

## 3. Results

### 3.1. Effects of 10% Changes in Channels Conductances on AP Morphology and Intracellular Ca^2+^ Dynamics

Figure 2 shows the effects of 10% changes in 19 default ion channel conductances and permeabilities (see the appendix, Table 1). Both PLS and Nimrod/E methods yielded consistent results with respect to single parameter's changes on the model outputs (APD_60_, Δ[Ca]_*i*_, Δ[Ca]_SL_, Δ[Ca]_jct_).

**Figure 3:**
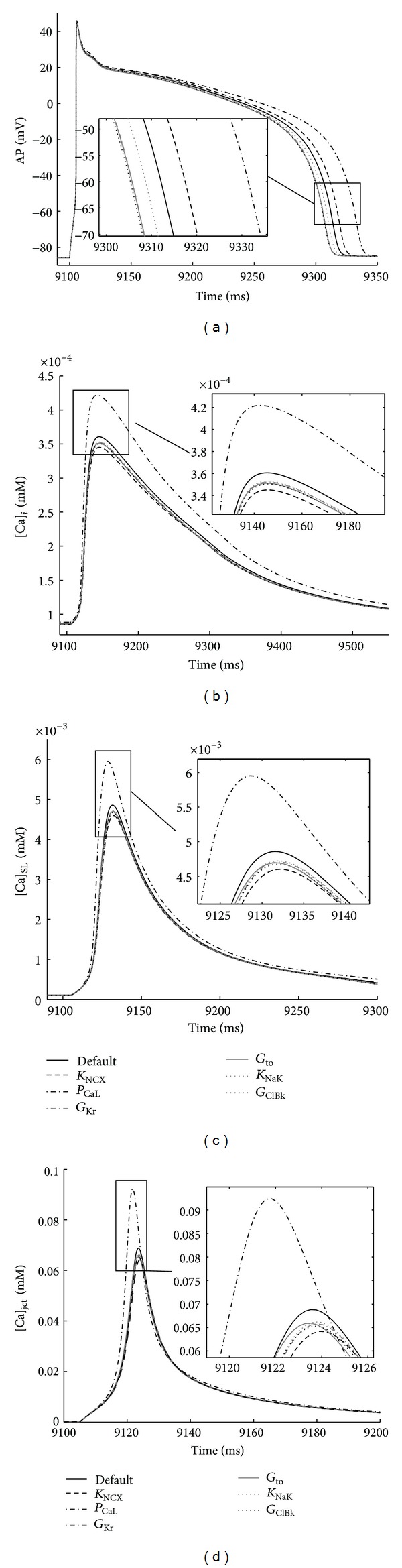
Verification of ion conductance sensitivity predictions. Steady-state AP and Ca^2+^ transients (recorded 9-10 s) in each of the three non-SR compartments in response to 1 Hz stimulus. The predicted changes in APD_60_ and Ca^2+^ peaks (see *Insets* also) are consistent with the generated by Nimrod/E and PLS outputs with respect to 10% increases in transporters' conductance. Control conductance values (black solid line) and 10% increases in *K*_NCX_ (black dashed line), *P*_CaL_ (black dash-dot line), *G*_Kr_ (gray dash-dot line), *G*_to_ (gray solid line), *K*_NaK_(gray dotted line), and *G*_ClBk_ (black dotted line).

The increase in L-type Ca^2+^ channel permeability (*P*_CaL_) had the most pronounced effects on APD_60_ and Ca^2+^ signals, by increasing action potential duration and enhancing maximum Ca^2+^ peaks. The effects of *G*_Kr_, *G*_to_, and *K*_NCX_, while less pronounced than *P*_CaL_, were still significant. Figure 2 also shows that 10% increases in *G*_Kr_ and *G*_to_ decreased APD_60_ and Ca^2+^ peaks while 10% increase in *K*_NCX_ decreased Ca^2+^ peaks but increased APD_60_. The increase in maximal NaK pump rate (*K*_NaK_) had still detectable but minor effect, by decreasing both APD_60_ and Ca^2+^ peaks. The changes in SR parameters (*K*_*rel*⁡_, *K*_up_, and *K*_leak_) by 10% had negligible effects on APD_60_ and Δ[Ca]_*i*_ whereas the submembrane and junctional Ca^2+^ peaks (e.g., Δ[Ca]_SL_ and Δ[Ca]_jct_) were most affected. Figure 2 shows that 10% increases in *K*_*rel*⁡_ and *K*_up_ increased Δ[Ca]_SL_ and Δ[Ca]_jct_ while *K*_leak_ had the opposite effect. Interestingly, 10% increase in background Cl^−^ conductance (*G*_ClBk_) showed similar magnitude as *G*_Kr_ and *G*_to_ and demonstrated the same effects in decreasing APD_60_ and Ca^2+^ peaks. Furthermore, Nimrod/E had the advantage in predicting how 10% increases in two-parameter group can affect the model outputs. Nimrod/E predicts here that *P*_CaL_ and K_NCX_ combined, as well all other two-parameter group combinations (not shown in Figure 2), had slight or no effect on the selected cellular biomarkers (APD_60_, Δ[Ca]_*i*_, Δ[Ca]_SL_, and Δ[Ca]_jct_). The plots in Figure 3 confirm further the specific, quantitative predictions made by PLS and Nimrod/E for the effects of 10% increases in *P*_CaL_, *K*_NCX_, *G*_Kr_, *G*_to_, *K*_NaK_, and *G*_ClBk_ on AP morphology and Ca^2+^ transients.

### 3.2. Effects of Changes in Membrane Transporter Distributions on AP Morphology and Intracellular Ca^2+^ Dynamics

In Shannon et al. model, cell was separated into four lumped compartments (see Figure 1): the junction junctional cleft (0.077% of total cell volume assuming 11% of the surface membrane junctional), the subsarcolemmal space (2% of total cell volume assuming 89% of the membrane nonjunctional) the bulk cytosol space (65% of total cell volume with remainder of the volume accounted for by mitochondria) and the SR (3.5% of total cell volume). The L-type Ca^2+^ channels were assumed concentrated within the junctional membrane such that 90% of total number were located there while all other membrane transporters (Na^+^ channels, Na^+^ leak, Na^+^/K^+^ pump, slow delayed rectified K^+^ channel, Ca^2+^ activated Cl^−^ channel, Ca^2+^ leak, Na^+^/Ca^2+^ exchanger, and sarcolemmal Ca^2+^ pump) were considered evenly distributed across the sarcolemma with 11% in the junctional cleft and 89% in the subsarcolemmal compartment [1].

**Figure 4:**
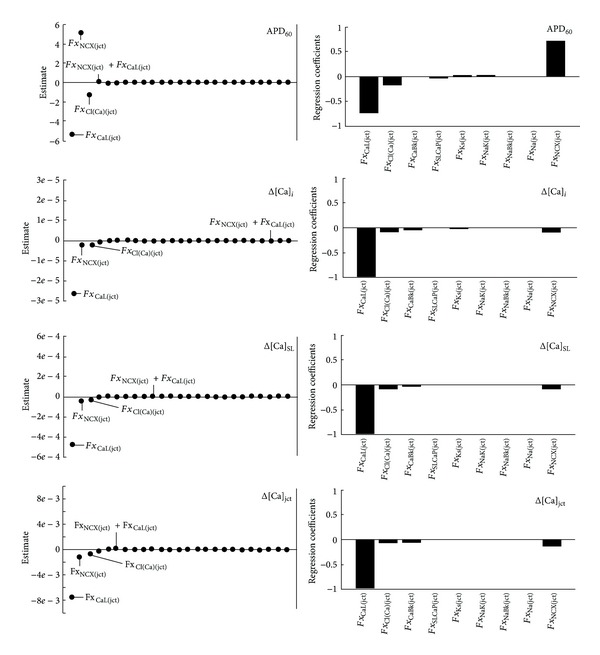
Parameter sensitivity analysis of APD_60_, Δ[Ca]_*i*_, Δ[Ca]_SL_, and Δ[Ca]_jct_ sensitivities toward 5% increases in ion transporter distributions in the junctional cleft. Transporters' distribution between sarcolemma and junctional cleft summed up to 100%. Increasing fraction of total *j-*current in the junction by 5% is analogous to decreasing fraction of total *j-*current in the subsarcolemma by 45% for *I*_Ca_ and by 40-41% for all other currents. Positive value indicates direct relationship between distribution and output (e.g., increased density of *I*_Ca_ in the junctional cleft shortens APD_60_) and negative value indicates indirect relationship between parameter and output. Nimrod/E-Lenth plots (left column), PLS-bar plots (right column). Nimrod/E analysis produced similar qualitative results as PLS analysis.

**Figure 5:**
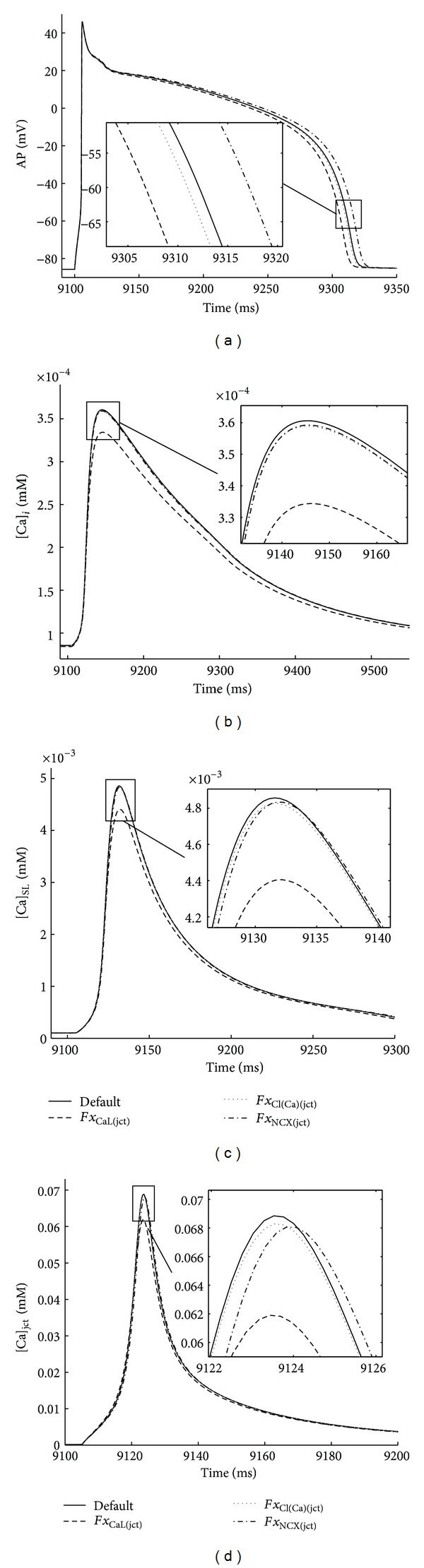
Verification of transporter distribution sensitivity predictions. Steady-state model outputs (recorded 9-10 s) in response to 1 Hz periodic pulse. The predicted alterations in APD_60_ and Ca^2+^ peaks (see *Insets* also) are consistent with the generated by Nimrod/E and PLS results with respect to 5% increases in transporters' fraction in the junctional cleft. Default *j*-currents' fractions in junctional cleft (black solid line) and 5% increases in *Fx*_CaL(jct)_ (black dashed line), *Fx*_Cl(Ca)(jct)_ (grey dotted line), *Fx*_NCX(jct)_ (black dash-dot line).

To examine how the changes in ion transporter distributions affect the selected cellular biomarkers (APD_60_, Δ[Ca]_*i*_, Δ[Ca]_SL_, and Δ[Ca]_jct_), we performed systematic analysis, increasing by 5% the basic *Fx*_*j*(jct)_ while decreasing *Fx*_*j*(SL)_ to keep total number of transporter protein complexes within the sarcolemma unchanged; that is, *Fx*_*j*(jct)_ + *Fx*_*j*(SL)_ = 1 (the appendix, Table 2).  Figure 4 shows that PLS and Nimrod/E analysis again showed consistent results. The 5% increase in L-type Ca^2+^ channels fraction in the junctional cleft (*Fx*_CaL(jct)_ = 0.945) had the most pronounced effect on the model outputs, decreasing APD_60_ and Ca^2+^ peaks. The results also suggest that 5% increase in the junctional Na^+^-Ca^2+^ current (*Fx*_NCX(jct)_ = 0.1155) prolonged APD_60_ while slightly affected Ca^2+^ peaks in the junctional cleft and subsarcolemmal and bulk cytosol compartments. The 5% increase in the junctional *I*_Cl(Ca)_ (*Fx*_Cl(Ca)(jct)_ = 0.1155) had similar effect on all model outputs as the increase in *Fx*_CaL(jct)_ but with much smaller magnitude. Results in Lenth plot (Figure 4) for the four model outputs reveal that “*Fx*_NCX(jct)_ *Fx*_CaL(jct)_ combination” as well all other two-parameter combinations had insignificant impact (not shown in Figure 4). Additional analysis of the data has been done by increasing each *Fx*_*j*(jct)_ by 5% and decreasing *Fx*_*j*(SL)_ (*j* = CaL, NCX, Cl(Ca)) while keeping all other model parameters constant. The results in Figure 5 confirm the predictions made by both PLS and Nimrod/E methods.

### 3.3. Effects of 10% Changes in Diffusion Constants on AP Morphology and Intracellular Ca^2+^ Dynamics

**Figure 6:**
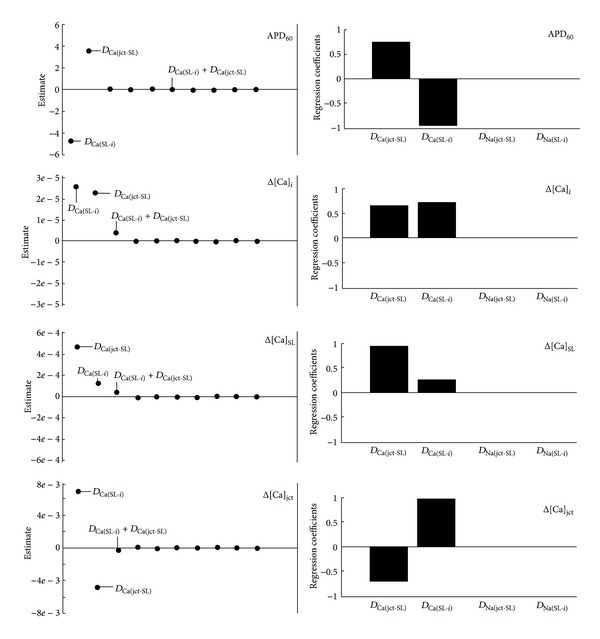
Parameter sensitivity analysis of APD_60_, Δ[Ca]_*i*_, Δ[Ca]_SL_, and Δ[Ca]_jct_ sensitivities toward 10% increases in Ca^2+^ and Na^+^ diffusion coefficients between subcellular compartments. Positive value indicates direct relationship between diffusion coefficient and output (e.g., increased diffusion coefficient between junctional cleft and sarcolemma prolongs APD_60_) and negative sensitivity value indicates indirect relationship between parameter and output. Nimrod/E-Lenth plots (left column), PLS-bar plots (right column). Nimrod/E analysis produced similar qualitative results as PLS analysis.

**Figure 7:**
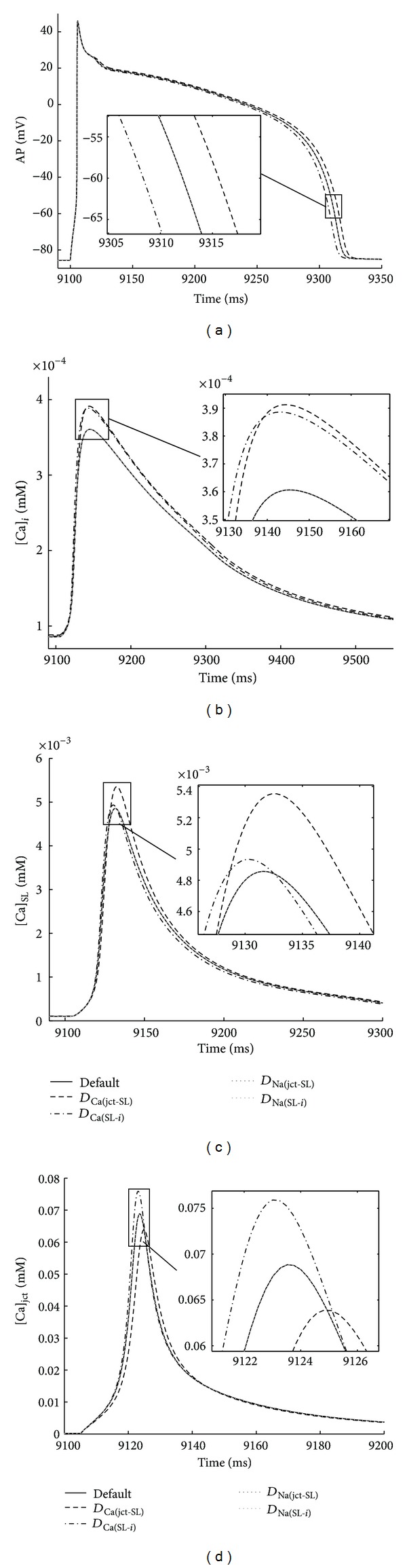
Verification of Ca^2+^ and Na^+^ diffusion sensitivity predictions. Steady-state AP and Ca^2+^ transients (recorded 9-10 s) in three non-SR compartments in response to 1 Hz stimulus. The relative changes in APD_60_ and Ca^2+^ peaks (see *Insets* also) are consistent with predictions generated by Nimrod/E and PLS with respect to 10% increases in Ca^2+^ and Na^+^ diffusion constants. Control diffusion coefficients (black solid line) and 10% increases in *D*_Ca(jct-SL)_ (black dashed line), *D*_Ca(SL-*i*)_ (black dash-dot line), *D*_Na(jct-SL)_ (gray dotted lines), *D*_Na(SL-*i*)_ (light gray dotted lines).

The effects of changes in default parameters describing Ca^2+^ and Na^+^ diffusion between junctional and subsarcolemmal compartments and between subsarcolemmal and bulk compartments were also analyzed in this study (the appendix, Table 3).  Figure 6 shows that 10% increases in basal *D*_Ca(jct-SL)_ altered all model outputs, prolonging APD_60_ and increasing Δ[Ca]_*i*_ and Δ[Ca]_SL_ but decreasing Δ[Ca]_jct_. Moreover 10% increases in basal *D*_Ca(SL-*i*)_ value shortened APD_60_ and increased Δ[Ca]_*i*_, Δ[Ca]_jct_, and Δ[Ca]_SL_. Interestingly, 10% increases in basal *D*_Na(jct-SL)_ and *D*_Na(SL-*i*)_ values in all three compartments had no effect on APD_60_ and Ca^2+^ peaks. Nimrod/E analysis also suggests that two-level interaction between *D*_Ca(jct-SL)_ and *D*_Ca(SL-*i*)_ had a minor effect on increasing Δ[Ca]_*i*_ while APD_60_, Δ[Ca]_SL_, and Δ[Ca]_jct_ were unaffected.  Figure 7 additionally confirms the PLS and Nimrod/E predictions for the effects of 10% increases in *D*_Ca(jct-SL)_ and *D*_Ca(SL-*i*)_ on AP morphology and Ca^2+^ transients.

## 4. Discussion

The main goal of this study was to perform a systematic investigation on how small variations in ionic currents and Ca^2+^ and Na^+^ diffusion coefficients modulate ventricular myocyte electrophysiology and Ca^2+^ dynamics in rabbit ventricular myocytes. To determine the most influential parameters controlling underlying subcellular mechanisms, we applied and compared the outcomes from two sensitivity analysis toolkits (PLS and Nimrod). The sensitivity of selected biomarkers (APD_60_, Δ[Ca]_*i*_, Δ[Ca]_SL_, and Δ[Ca]_jct_) to the alterations in ionic currents' properties and intracellular ionic transport was quantified using the Shannon et al. ionic model in rabbit ventricular cells, the predictive power of which has been demonstrated previously [1, 2].

### 4.1. Effects of Small Variations in Ionic Currents on AP Duration and Ca^2+^ Transients

Using parameter sensitive analysis, Romero et al. quantified the sensitivity of AP properties, Ca^2+^ and Na^+^ dynamics, and their rate dependence with respect to ±15%, ±30%, ±45%, and ±100% variations in the main transmembrane currents' conductance [9]. It remains, however, poorly understood how *small variations* (±5, ±10%) in ion currents conductance affect cellular biomarkers. Our next studies contribute to filling up this knowledge gap further. Analysis suggests that the selected biomarkers were strongly dependent on *I*_Ca_ current properties. Results indicate that 10% increase in maximal permeability of L-type Ca^2+^ channel (*P*_CaL_), with respect to other currents' conductance changes, most notably affected APD_60_ and Ca^2+^ transients' peaks at 1 Hz. Our predictions for the effects of *P*_CaL_ on APD and cytosolic Ca^2+^ peak are also consistent with reported data in rabbit ventricular myocytes after applications of *I*_Ca_ blockers [5, 9, 29] or in cells with high L-type Ca^2+^ channel density [30]. The effects of 10% increase in *G*_Kr_, *G*_to_, *K*_NCX_, or *K*_NaK_ on cellular biomarkers, while less pronounced than *P*_CaL_, were still detectible. Our predictions for *K*_NCX_ increase at 1 Hz are in qualitative agreement with data in ferret myocytes [31] and data supporting APD prolongation following *I*_NCX_ block reported in some experiments in rabbit cardiomyocytes [32, 33]. Several studies in rabbit ventricular cells report also a strong effect of *I*_NCX_ in modulating systolic [Ca]_*i*_ transient [31–34] while others report only a moderate effect [35], similar to our predictions for Δ[Ca]_*i*_. Results demonstrate also that 10% increase in *K*_NCX_ slightly decreased [Ca]_jct_ and [Ca]_SL_ peaks. Increase in *G*_Kr_ and *G*_to_ at 1 Hz resulted in APD shortening, which is consistent with experiments in rabbit ventricular myocytes [36, 37]. Important role of *I*_to_ in modulating Ca^2+^ transients through its effects on *I*_Ca_ and *I*_NCX_ during AP repolarization period has been suggested [38, 39]. Slight effects, however, due to 10% increase in *G*_to_ at 1 Hz on Ca^2+^ peaks are predicted here. Furthermore, at stimulus frequency of 2.5 Hz, Romero et al. report *I*_NaK_ (±30% changes or severe *I*_NaK_ block; 0–8 min) as important influent current in most simulated cellular biomarkers, including APD_60_ [9]. Our studies quantitatively confirmed Romero et al. findings at 2.5 Hz (data not shown). At 1 Hz, however, the effect of ±30% changes in *K*_NaK_ (increment 5%, 0–8 min) on APD_60_ was less pronounced. The main reason for this observation was that the predicted changes in [Na]_*i*_ were smaller at 1 Hz (20–100 *μ*M) versus 1–1.5 mM at 2.5 Hz, in this way affecting differently *I*_NaK_ and *I*_NCX_ activities. These results indicate that the stimulus frequency is important factor regulating the electrophysiological biomarkers. Accordingly, because the Shannon et al. model mimics rabbit electrophysiology more accurately at normal pacing rates of 1 Hz [9], the model predictions for the effects of changes of ion current conductivities at faster rates should be considered with caution. Interestingly, 10% increase in Cl^−^ background current (*I*_ClBk_), which is a new feature of the Shannon et al. model, showed a negative relationships with four outputs (APD_60_, Δ[Ca]_*i*_, Δ[Ca]_SL_, and Δ[Ca]_jct_) of approximately similar magnitude as for *G*_to_ or *G*_Kr_. Experimental data reporting the effects of *I*_ClBk_ on rabbit ECC are missing in the literature however. Another interesting finding is the relatively small sensitivity of the biomarkers to 10% increases in *G*_Na_, *G*_Na,b_, *P*_CaK_, *P*_CaNa_, *G*_Ca,b_, *G*_to,f_, *G*_Ks_, *G*_K1_, *G*_Cl(Ca)_, *K*_*rel*⁡_, *K*_leak_, *K*_up_, and *K*_pCa_ factors at 1 Hz. Thus, to further test the predictive power of the Shannon et al. model we examined the effects of severe currents' and SR-fluxes' block (up to 100%) (data not shown). In qualitative agreement with experiment [9] our studies show that (1) APD_60_ was highly sensitive to 100% *I*_Na_, *I*_K1_, *K*_*rel*⁡_, and *K*_up_ and block and less sensitive to *I*_CaK_, *I*_Cl(Ca)_, *I*_to,f_, and *I*_pCa_ inhibition while 100% block of *I*_Na,b_, *I*_CaNa_, *I*_Ca,b_, and *I*_Ks_ currents and SR Ca^2+^ leak (*K*_leak_) had small or no effect; (2) steady-state Ca^2+^ peaks were strongly affected when *I*_Na_, *I*_Ca,b_, and SR Ca^2+^ fluxes (*K*_*rel*⁡_, *K*_leak_, and *K*_up_) were inhibited whereas 100% block *I*_pCa_, *I*_Cl(Ca)_, *I*_CaK_, *I*_K1_, and *I*_to,f_ had less pronounced effects. New measurements in rabbit ventricular myocytes are needed to define the relative importance of *G*_Na_, *G*_Na,b_, *P*_CaK_, *P*_CaNa_, *G*_Ca,b_, *G*_to,f_, *G*_Ks_, *G*_K1_, *G*_Cl(Ca)_, *K*_*rel*⁡_, *K*_leak_, *K*_up_, and *K*_pCa_ in determining physiological responses due to the small changes in these factors at 1 Hz and to test further the capabilities and limitations of the Shannon et al. model. Using Nimrod/E, we also examined how simultaneous changes in two transmembrane currents affect APD and Ca^2+^ dynamics. Our analysis suggests that 10% increase in both *P*_CaL_ and *K*_NCX_ had minor effect at 1 Hz. Additionally, Nimrod/E showed that simultaneous increases in control *I*_Ca_ and K^+^ related currents had slight or no effect (data not shown). Furthermore, the predicted effects of ±5% changes in channels conductance were identical to those with ±10% variations (data not shown). Such insights, coming from mathematical models, might be useful in searching new avenues for development of methodologies to predict drug action effects on behavior of cardiac cells. For example, most *I*_Ca_ inhibitors suppress partially K^+^-related currents also; therefore the balance of these currents is critical for predicting *I*_Ca_-related drug action.

### 4.2. Effects of Variations in Membrane Transporters Distribution on AP Duration and Ca^2+^ Transients

Experimental studies have demonstrated that in rabbit ventricular myocytes marked variations in the distribution of ion transports along the surface membrane probably exist [1, 40–42]. Using reaction-diffusion models, recently we demonstrated that the subcellular Ca^2+^ signals are highly sensitive to *I*_Ca_ and *I*_NCX_ fluxes distributions via the sarcolemma [13, 16, 19, 20]. In these spatial models, however, the cell electrophysiology was simplified; equations describing *I*_Ca_, *I*_NCX_, *I*_pCa_, and *I*_Ca,b_ properties were only included; and AP dynamics were not reproduced. Additionally, *small variations* in L-type Ca^2+^ channels density in junctional cleft have been suggested to lead to large changes in control SR Ca^2+^ release, subcellular Ca^2+^ transients, and AP waveform [4, 5]. Thus, we used PLS and Nimrod/E to gain further insights into how ±5%* changes* in L-type and other ion transporters' distributions influence control AP and Ca^2+^ transients. We evaluated the biomarkers' sensitivity to 5% increases in the fraction of total ionic current in the junctional cleft (*Fx*_*j*(jct)_) assuming total number of transporters within the sarcolemma unchanged; that is, *Fx*_*j*(jct)_ + *Fx*_*j*(SL)_ = 1. Our results yielded three channel distributions (*Fx*_CaL(jct)_, *x*_NCX(jct)_, and *Fx*_Cl(Ca)(jct)_) that significantly affect the model outcomes of interest (i.e., APD_60_, Δ[Ca]_*i*_, Δ[Ca]_SL_, and Δ[Ca]_jct_). Increasing by 5% the density of L-type Ca^2+^ channels in the junctional cleft (while decreasing by 45% *I*_Ca_ density in SL compartment to keep *Fx*_CaL(jct)_ + *Fx*_CaL(SL)_ = 1) had the strongest effect on both APD_60_ and Ca^2+^peaks, decreasing APD_60_ and [Ca]_*i*_, [Ca]_SL_, and [Ca]_jct_ peaks. The reason for the predicted decrease in [Ca]_jct_ was the strongly increased rate of Ca^2+^ transfer from the junctional cleft to the submembrane space evoked by the 5% increase of *Fx*_CaL(jct)_ and reduced *I*_Ca_ (~45%) into the SL compartment. The predicted decreases into [Ca]_SL_ peak and APD_60_ were also consequence of *Fx*_CaL(SL)_ reduction to 45% though Ca^2+^ flux from the cleft was increased. The drop in [Ca]_SL_ evoked decrease in the rate of Ca^2+^ transfer from the SL to cytosolic compartment thus causing drop in [Ca]_*i*_ peak as well. Our studies also showed that the effects of *I*_NCX_ redistribution were quite different. The 5% increase in *Fx*_NCX(jct)_ (while decreasing by 40-41% *I*_NCX_ density in SL compartment to keep *Fx*_NCX(jct)_ + *Fx*_NCX(SL)_ = 1) prolonged APD_60  _to approximately the same magnitude as for *I*_Ca_ and slightly decreased the control Ca^2+^ peaks. Significant effects due to *I*_Ca_ redistribution and negligible effects due to *I*_NCX_ redistribution on the local and global Ca^2+^ transients' peaks were also found using our reaction-diffusion model in rabbit ventricular cells [20]. Calcium activated chloride current (*I*_Cl(Ca)_) has been extensively studied [43–45]. Experimental data in rabbit ventricular myocytes suggest that *I*_Cl(Ca)_ is strongly temperature dependent (very small at room temperature but substantial at 35°C–37°C); *I*_Cl(Ca)_ can be eliminated by blocking *I*_Ca_; and *I*_Cl(Ca)_ may normally facilitate rapid repolarization when SR Ca^2+^ load and release are high. To bring further insights into the effects of *I*_Cl(Ca)_ properties on ECC, we examined how 5% increase in *I*_Cl(Ca)_ density in the junctional cleft (assuming *Fx*_Cl(Ca)(jct)_ + *Fx*_Cl(Ca)(SL)_ = 1) may affect the cell electrical activity and Ca^2+^ signaling. Interestingly, although much less pronounced than *Fx*_CaL(jct)_ variations, changes in *Fx*_Cl(Ca)(jct)_ were able also to shorten APD_60_  and provide negative feedback on cell Ca^2+^ load. Negligible effects due to 5% increases in *I*_Na_, *I*_Na,b_, *I*_NaK_, *I*_Ks_, *I*_Ca,b_, and *I*_pCa_ currents' fractions in the junctional cleft were found. Finally, Nimrod/E predicted slight or no effect on model outputs when *Fx*_CaL(jct)_ and *Fx*_NCX(jct)_, or any other two-parameter *Fx*_j(jct)_ groups, were combined. These model studies imply that more accurate experimental knowledge of *I*_Ca_, *I*_Cl(Ca)_ and *I*_NCX_, fluxes distributions is needed to better understand the physiological role of *I*_Cl(Ca)_ current, the local Ca^2+^ signaling, and the electrical cell activity. Furthermore, the estimates of % total flux in junctional cleft versus submembrane space under control conditions are still under debate. Additional quantification of the model sensitivity to ±10% changes in *Fx*_CaL(jct)_ or ±10% and ±15% variations in other transporters' densities in the cleft (not done in this study) may provide a more convincing conclusions.

### 4.3. Effects of Small Variations in Intracellular Ion Transport on AP Duration and Ca^2+^ Transients

In the Shannon et al. model the diffusion of Ca^2+^ and Na^+^ ions was considered where (1) the junctional compartment communicates only with the SL compartment, (2) the SL compartment with the junctional and the bulk cytosolic compartments, and (3) the bulk cytosolic compartment only with the SL compartment. The values of the effective diffusion coefficients for Ca^2+^ and Na^2+^ were average values measured in different cardiac tissues and species under control conditions. In the literature, however, these estimates are contradictable [1, 8, 10–20]. In this study, we tested how *small changes* (±5, ±10%) in suggested diffusion coefficients may influence the model outputs. We used PLS and Nimrod toolkits to determine the most influential ion diffusion parameter(s) governing the properties of the electrical signal (such as APD_60_) and of the Ca^2+^ signal (such as peak level of Ca^2+^ transient in the junctional cleft, subsarcolemmal space, and bulk cytosol). While 10% increases in basal Ca^2+^ diffusion coefficients (*D*_Ca(jct-SL)_ and *D*_Ca(SL-*i*)_) had pronounced effects on the model outputs, no visible changes in all selected biomarkers were detected due to 10% increases in Na^+^ diffusion coefficients (*D*_Na(jct-SL)_ and *D*_Na(SL-*i*)_). Interestingly marked effects on both APD_60_ and Ca^2+^ peaks were predicted when Na^+^ diffusion was blocked, assuming *D*_Na(jct-SL)_and *D*_Na(SL-*i*)_ zeros shorten APD_60_ and decreased [Ca]_*i*_, [Ca]_SL_, and [Ca]_jct_ peaks (data not shown). Our results also demonstrate that accelerating Ca^2+^ transfer from junctional to submembrane space (e.g., increasing *D*_Ca(jct-SL)_), (1) sensitively decreased Ca^2+^ peak in the junctional cleft and prolonged [Ca]_jct_ time to peak, (2) enhanced [Ca]_SL_ and [Ca]_*i*_ peaks, and (3) prolonged APD_60_. The effects of *D*_Ca(SL-*i*)_ increase were quite different. Accelerating Ca^2+^ transfer from the SL compartment to the bulk cytosol (e.g., increasing *D*_Ca(SL-*i*)_) shortened APD_60_ and enhanced all Ca^2+^ transients' peaks. In addition, *D*_Ca(SL-*i*)_ increase most significantly enhanced [Ca]_jct_ peak while the control [Ca]_SL_ peak was less affected. Possible reason for the predicted increase in [Ca]_jct_ peak, due to *D*_Ca(SL-*i*)_ increase, was enhanced [Ca]_*i*_ which promoted increase in the SR load (i.e., [Ca]_SR_) in this way causing additional Ca^2+^ release from the SR and consequent elevations in [Ca]_jct_ and [Ca]_SL_, respectively. The enhanced [Ca]_SL_ transient produced faster Ca^2+^-dependent inactivation of *I*_Ca_, thus leading to APD shortening. Nimrod/E showed also that simultaneous increases in control *D*_Ca(jct-SL)_ and *D*_Ca(SL-*i*)_ slightly increased Δ[Ca]_*i*_ while all other biomarkers remained insensitive to the changes in any other two-level parameter combinations. Also predicted relative effects of ±5% changes in intracellular ion transport were identical to those of ±10% variations (data not shown). In summary, our results reveal that small variations in diffusion coefficients for Ca^2+^ may impact sensitively the cell electrical activity and Ca^2+^ homeostasis. New and more precise estimates of the effective Ca^2+^ and Na^+^ diffusion constants are needed to better understand ECC in rabbit ventricular myocytes under control and certain pathologies.

We need to acknowledge that although we demonstrated good correlation with experiment, the results of sensitivity analysis strongly depend on the model details and simplifications. For example, in the lumped Shannon et al. model the ionic diffusion from junctional cleft to cytosolic compartment goes solely through subsarcolemmal space which is questionable with regard to the contact areas between these compartments [19]. In addition, in all studied cases the variations of intracellular potassium concentration were disabled (i.e., *d*[K]_*i*_/*dt* = 0 in Shannon's MatLab code) and whether or not this drawback was eliminated in the Romero's code or in recent modifications of the Shannon's code is unclear [4–7, 9]. This limitation leaded to the following: (1) fast stabilisation of the model for [Ca]_*i*_ and AP (after 6–8 s until 8 min); (2) [Na]_*i*_ appeared stable after ~200 s; (3) diastolic [K]_*i*_ remained unchanged in the interval 0 ≤ *t* ≤ 8 min. Thus, models neglecting slow intracellular concentration changes in [K]_*i*_ produced mainly by Na/K pump need to be revised further. Finally, the *t*-tubule compartment, the ion diffusion between the *t*-tubule lumen and bulk extracellular space, and the mitochondrial Ca^2+^ fluxes were omitted [46–50]. These are likely to have important impact on predicted AP and Ca^2+^ profiles as well.

## 5. Conclusions 

Our studies demonstrate that parameter sensitivity approaches can be used to obtain new insights into relationships between model parameters, model outputs, and experimental data. More specifically, the simulation results provided information on how small variations in the ionic currents and intracellular Ca^2+^ and Na^+^ diffusion modulate ventricular myocyte electrophysiology and Ca^2+^ dynamics in rabbit ventricular myocytes at 1 Hz. We found that APD_60_ and Ca^2+^ peaks are (1) highly sensitive to 10% increase in *I*_Ca_, while the effects of *I*_NCX_, *I*_NaK_, *I*_Kr_, *I*_to_, and *I*_ClBk_ were moderate, and (2) insensitive to 10% increases in all other Ca^2+^, Na^+^, and K^+^ transmembrane currents and SR Ca^2+^ fluxes. The myocyte electrical activity is highly sensitive to 5% variations in *I*_Ca_ and *I*_NCX_ and less sensitive to *I*_Cl(Ca)_ redistribution between junctional cleft and submembrane space. Small changes in submembrane and cytosolic diffusion coefficients for Ca^2+^, but not in Na^+^ diffusion coefficients, may impact notably the myocyte contraction. This study highlights the need for additional and more precise experimental data and further updating and testing of the Shannon et al. model and its recent modifications to fill specific knowledge gaps related to rabbit ventricular electrophysiology and intracellular Ca^2+^ signaling.

## Figures and Tables

**Table 1:** Maximal conductance varied in the model.

Definition		Symbol	Baseline value
1	Maximal Na^+^ conductance	*G*_Na_	16 mS/μF
2	Background Na^+^ conductance	*G*_Nab_ (*G*_NaBk_)	0.297 10^−3^ mS/*µ*F
3	Maximal rapid delayed rectifier K^+^ conductance	*G*_Kr_ (*G*_IKr_)	0.07 mS/*µ*F
4	Maximal slow delayed rectifier K^+^ conductance	*G*_Ks_	0.013 mS/*µ*F
5	Maximal fast transient outward K^+^ conductance	*G*_to,*f*_	0.06 mS/*µ*F
6	Maximal slow transient outward K^+^ conductance	*G*_to_ (*G*_to, *s*_)	0.02 mS/*µ*F
7	Maximal inward rectifier K^+^ conductance	*G*_K1_	0.9 mS/*µ*F
8	Maximal Ca-activated Cl^−^ conductance	*G*_Cl(Ca)_	0.109625 mS/*µ*F
9	Background Cl^−^ conductance	*G*_ClBk_	0.009 mS/*µ*F
10	Maximal permeability of L-type Ca^2+^ channel for Ca^2+^	*P*_CaL_ (*P*_Ca_)	5.4 10^−4^ cm/s
11	Maximal permeability of L-type Ca^2+^ channel for Na^+^	*P*_CaNa_ (*P*_Na_)	1.5 10^−8^ cm/s
12	Maximal permeability of L-type Ca^2+^ channel for K^+^	*P*_CaK_ (*P*_K_)	2.7 10^−7^ cm/s
13	Background Ca^2+^ conductance	*G*_Cab_ (*G*_CaBk_)	0.0002513 ms/*µ*F
14	Maximal Na^+^-Ca^2+^ exchange current	*K*_NCX_ (${\bar{I}}_{\text{NCX}}$)	9 A/F
15	Maximal sarcolemmal Ca^2+^ pump current	*K*_pCa_ (${\bar{I}}_{\text{SLCaP}}$)	0.0673 A/F
16	Maximal Na^+^-K^+^ pump current	*K*_NaK_ (${\bar{I}}_{\text{NaK}}$)	1.91 A/F
17	SR Ca^2+^ release scaling factor	*K*_*rel*⁡_ (*k*_*s*_)	25 ms^−1^
18	Maximal rate of SR Ca^2+^ uptake (SERCA)	*K*_up_ (${\bar{I}}_{\text{SRCaP}}$)	5.3 10^−3^ mM/ms
19	Passive SR Ca^2+^ leak scaling factor	*K*_leak_ (*K*_SRleak_)	5.348 10^−6^ ms^−1^

Note: notations in brackets, for example, (…), show corresponding notations of some parameters used in Shannon et al. article [1]. The baseline conductance values used in this study are the same as in [1].

**Table 2:** Fractions of total “*j*” flux varied in the model.

Definition		Symbol	Baseline value
1	Fraction of total Na^+^ current in junctional cleft	*Fx*_Na(jct)_	0.11
2	Fraction of total background Na^+^ current in junctional cleft	*Fx*_NaBk(jct)_	0.11
3	Fraction of total Na^+^-K^+^ current in junctional cleft	*Fx*_NaK(jct)_	0.11
4	Fraction of total slow delayed rectifier K^+^ current in junctional cleft	*Fx*_Ks(jct)_	0.11
5	Fraction of total Ca-activated Cl^−^ current in junctional cleft	*Fx*_Cl(Ca)(jct)_	0.11
6	Fraction of total Ca-dependent L-type Ca^2+^ current in junctional cleft	*Fx*_CaL(jct)_	0.9
7	Fraction of total background Ca^2+^ current in junctional cleft	*Fx*_CaBk(jct)_	0.11
8	Fraction of total Na^+^-Ca^2+^ current in junctional cleft	*Fx*_NCX(jct)_	0.11
9	Fraction of total sarcolemmal Ca^2+^ pump current in junctional cleft	*Fx*_SLCaP(jct)_	0.11

Notes: fraction of total *j* flux in SL (*Fx*_*j*(SL)_ = 1 − *Fx*_*j*(jct)_). In the Shannon et al. article [1]  *Fx*_Cl(jct)_  corresponds to  *Fx*_Cl(Ca)(jct)_. The baseline total “*j*” flux fraction values used in this study are the same as in [1].

**Table 3:** Diffusion coefficients varied in the model.

Definition		Symbol	Baseline value
1	Diffusion coefficient for Ca^2+^ from junctional cleft to SL compartment	*D*_Ca(jct-SL)_	1.64 10^−6^ cm^2^/s
2	Diffusion coefficient for Ca^2+^ from SL compartment to bulk cytosol	*D*_Ca(SL-*i*)_	1.22 10^−6^ cm^2^/s
3	Diffusion coefficient for Na^+^ from junctional cleft to SL compartment	*D*_Na(jct-SL)_	1.09 10^−5^ cm^2^/s
4	Diffusion coefficient for Na^+^ from SL compartment to bulk cytosol	*D*_Na(SL-*i*)_	1.79 10^−6^ cm^2^/s

Notes: in the Shannon et al. article [1] *D*_Ca(junction-SL)_ corresponds to**** *D*_Ca(jct-SL)_,**** *D*_Ca(SL-cytosol)_  to  *D*_Ca(SL-*i*)_,  *D*_Na(junction-SL)_  to *D*_Na(jct-SL)_, and *D*_Na(SL-cytosol)_ to *D*_Na(SL-*i*)_. The baseline diffusion coefficients values used in this study are the same as in [1].

## Appendix

See Tables 1, 2, and 3.

## Glossary

### Abbreviations

ECC: Excitation-contraction coupling
AP: Action potential
APD_60_: Action potential duration at 60% repolarization
SL: Sarcolemmal membrane
SR: Sarcoplasmic reticulum
RyR: Ryanodine receptor
SERCA2: SR Ca^2+^ ATPase pump
TnC: Troponin C
NIMROD: Parameter modeling toolkit for sensitivity analysis
PLS: Partial least square tool.

### Membrane Currents

*I*_Na_: Na^+^ current
*I*_Na,b_ (*I*_NaBk_): Na^+^ background current
*I*_Ca_ (*I*_CaL_): L-type Ca^2+^ channel current
*I*_Ca,b_ (*I*_CaBk_): Ca^2+^ background current
*I*_to,f_: Fast transient outward K^+^ current
*I*_to_ (*I*_to,s_): Slow transient outward K^+^current
*I*_Kr_: Rapid delayed rectifier K^+^ current
*I*_Ks_: Slow delayed rectifier K^+^ current
*I*_K1_: Inward rectifier K^+^ current
*I*_Cl(Ca)_: Ca^2+^ activated Cl^−^ current
*I*_ClBk_: Cl^−^ background current
*I*_NaK_: Na^+^-K^+^ pump current
*I*_NCX_: Na^+^-Ca^2+^ exchanger current
*I*_pCa_ (*I*_SLCaP_): Sarcolemmal Ca^2+^ pump current.

### Concentrations

[Ca]_*i*_: Cytosolic Ca^2+^ concentration
[Ca]_SL_: Subsarcolemmal Ca^2+^ concentration
[Ca]_jct_: Junctional cleft Ca^2+^ concentration
[Ca]_SR_: SR Ca^2+^ concentration
[Na]_*i*_: Cytosolic Na^+^ concentration
[K]_*i*_: Cytosolic K^+^ concentration.
